# Supplementary material for: Characterization of Flavor Compounds in Distilled Spirits: Developing a Versatile Analytical Method Suitable for Micro-Distilleries
Source: Foods. 2022 Oct 25;11(21):3358. doi: 10.3390/foods11213358 (PMC9656916; doi:10.3390/foods11213358)
Supplement: Supplementary file 1 [file foods-11-03358-s001.zip › foods-1893851-supplementary.pdf]

Supplementary Material

Table S1: Selected LLE conditions for the extraction of distilled spirits

Reference	Samples	Solvent	Salt		Number of extractions
			Type	% (w/v)	
[1]	Brandy	CH_2Cl_2	NaCl	5.1	1
[2]	Tsipouro	CH_2Cl_2	NaCl	3.3	1
[3]	Tequila	CH_2Cl_2	-		2
[4]	Whiskey	CH_2Cl_2	Na_2SO_4	10	2
[5]	Whiskey	CH_2Cl_2	-		3
[6]	Cognac	Pentane/CH_2Cl_2 50:50	NaCl	10	1
[7]	Rum	Pentane/Et_20 50:50	NaCl	18	3
[8]	Brandy	Pentane/Et_20 70:30 x2, then Et_20	NaCl	26.7	3
[9]	Rum	Et_20	-		3
[10]	Cachaça, rum	Freon 113 + ultrasound	-		2

Table S2: Selected SPE conditions for the extraction of distilled spirits

Reference	Phase	Elution solvent
[11]	C_{18}	CH_2Cl_2
[12]	C_{18}	CH_2Cl_2/pentane 1:2
[13]	C_{18}	CH_2Cl_2
[14]	C_{18}	MeOH
[15]	Ethylvinylbenzene-divinylbenzene copolymer (LiChrolut EN)	CH_2Cl_2
[16]	Ethylvinylbenzene-divinylbenzene copolymer (LiChrolut EN)	CH_2Cl_2
[17]	Ethylvinylbenzene-divinylbenzene copolymer (LiChrolut EN)	CH_2Cl_2
[18]	N-vinylpyrrolidone-divinylbenzene copolymer (HLB)	Ethyl acetate/isooctane 80:20

Table S3: Selected SPME conditions for the extraction of distilled spirits

Reference	Samples	%EtOH (v/v)	%NaCl (w/v)	Fiber	T (°C)	Time (min)
[19]	Rum	12	18	PDMS	30	35
[20]	Rum	12	20	PDMS	30	35
[21]	Rum	-	-	PDMS	65	30
[22]	Whiskey	40	15	PDMS, DVB/CAR/PDMS	25	30
[23]	Whiskey	12	30	CAR/PDMS	40	60
[24]	Whiskey	13	30	CAR/PDMS	40	60
[25]	Gin	10	-	DVB/CAR/PDMS	50	30
[26]	Grain alcohol	20	-	DVB/CAR/PDMS	40	40
[27]	Rum, pear brandy, whiskey, vodka, grappa, egg liquor	-	-	DVB/CAR/PDMS	30	5
[28]	Banana terra spirit	3	25	DVB/CAR/PDMS	60	25
[29]	Gin, brandy, rum, vodka, whiskey, Chinese liquor	10	2	DVB/CAR/PDMS	50	15
[30]	Whiskey	20	-	PA	37	50
[31]	Cachaça, gin, vodka, whiskey, tequila	3	5	PA	60	25
[32]	Mezcal	-	-	CW/DVB	25	60

Table S4: Selected SBSE conditions for the extraction of distilled spirits

Reference	Samples	%EtOH (v/v)	%NaCl (w/v)	Phase	T (°C)	Time (min)
[33]	Whiskey	40	-	PDMS	25	30
[34]	Chinese liquor	10	20	PDMS	25	90
[35]	Sherry	20	-	PDMS	25	100

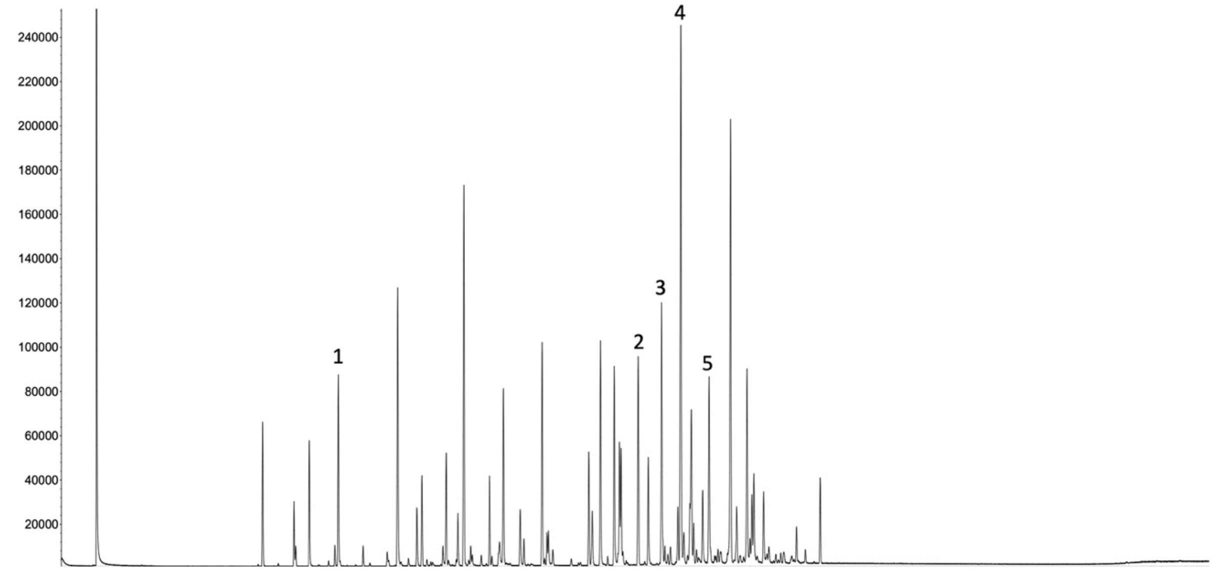

Figure S1: TIC chromatogram of 44°N gin (1μL, split 1:10): limonene **1**, β-caryophyllene **2**, α-humulene **3**, ɣ-muurolene **4**, δ-cadinene **5**

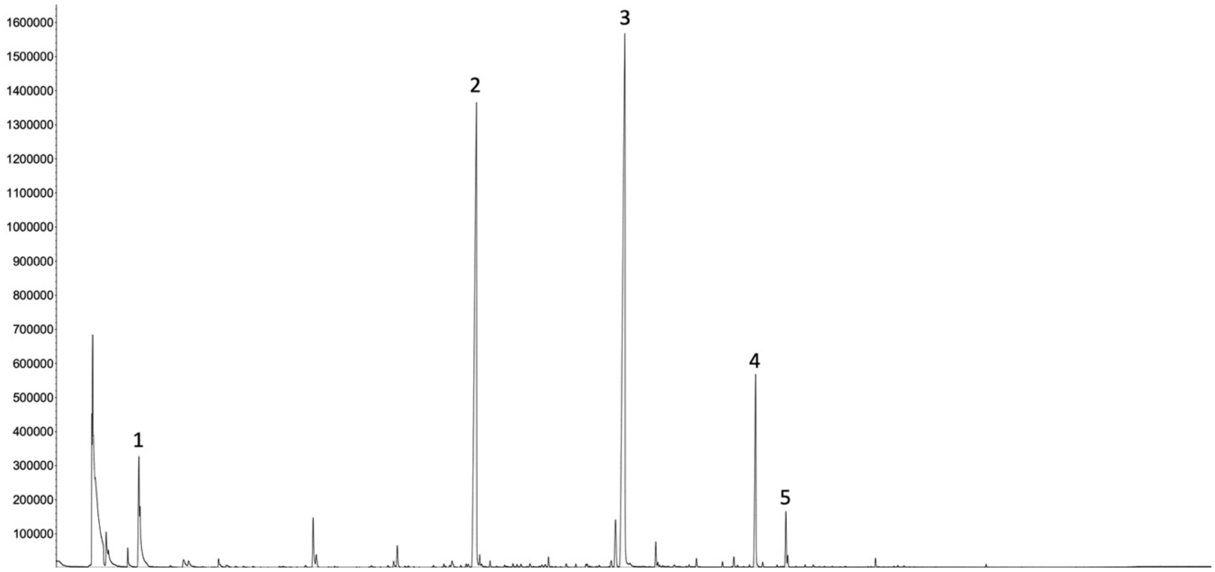

Figure S2: TIC chromatogram of Glenfiddich whiskey (1μL, split 1:10): 3-methyl-1-butanol **1**, ethyl octanoate **2**, 2-phenylethyl acetate **3**, ethyl decanoate **4**, ethyl dodecanoate **5**

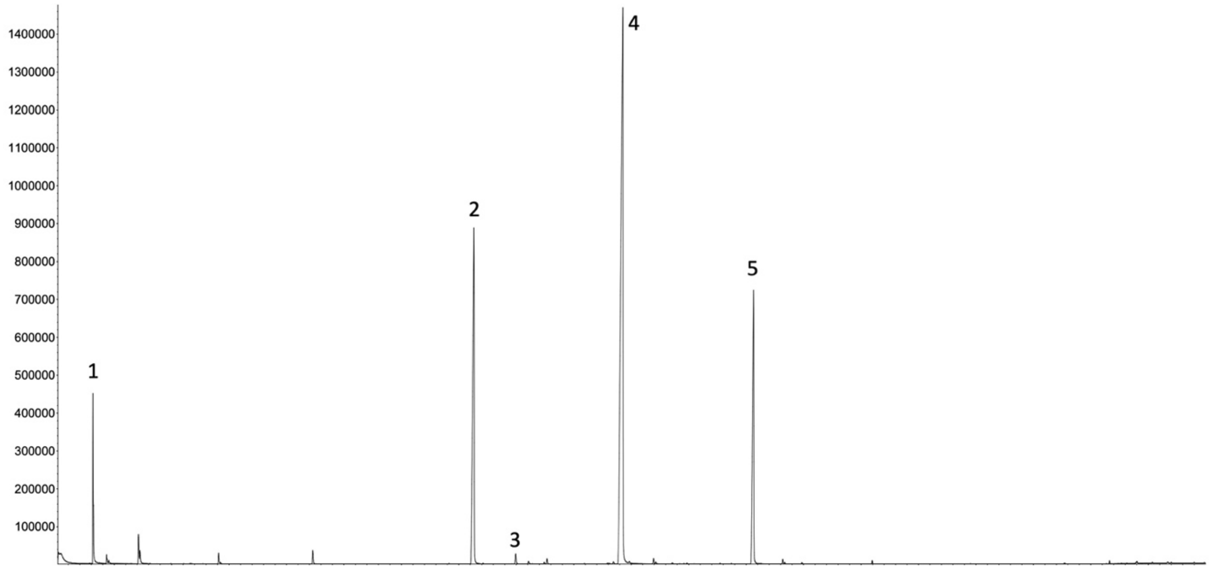

Figure S3: TIC chromatogram of Diplomatico rum (1μL, splitless): 3-methyl-1-butanol **1**, ethyl octanoate **2**, ethyl decanoate **3**, ethyl dodecanoate **4**, 3-methylbutyl decanoate **5**

Table S5: Most abundant m/z of unknown compounds with no identified chemical class in gin, whiskey and rum

Compound	Most abundant m/z (relative abundance %)
unknown 1	57(100); 56(99); 41(66); 39(8); 70(8); 58(6); 59(4); 53(3); 38(2); 51(2)
unknown 2	193(100); 209(44); 194(19); 195(15); 97(10); 135(10); 179(9); 210(8); 211(7); 165(5)
unknown 6	91(100); 119(100); 93(75); 180(74); 41(66); 109(64); 81(64); 107(54); 134(53); 121(53);
unknown 7	96(100); 55(99); 81(75); 97(62); 95(61); 67(60); 41(59); 69(48); 110(47); 68(34); 184(14)
unknown 9	157(100); 142(59); 141(39); 172(25); 115(23); 128(15); 158(11); 143(10); 155(9); 129(9); 170(4)
unknown 12	79(100); 41(70); 96(67); 109(52); 123(51); 93(49); 91(48); 81(45); 95(44); 107(43); 235(2)
unknown 13	131(100); 123(99); 91(87); 105(68); 124(66); 159(56); 109(54); 145(51); 119(44); 146(42); 220(88)
unknown 15	93(100); 69(90); 41(78); 107(55); 91(48); 119(41); 55(40); 105(38); 161(36); 67(35); 204(13)
unknown 21	157(100); 142(55); 172(42); 128(32); 141(30); 115(28); 129(24); 132(21); 158(20); 143(18); 218(5)
unknown 22	187(100); 123(84); 202(82); 159(63); 91(58); 109(51); 124(47); 131(42); 93(41); 41(40); 341(16)
unknown 25	69(100); 81(83); 41(74); 67(53); 95(51); 123(48); 55(43); 57(36); 91(36); 43(32); 181(18)

[1] J. Ledauphin *et al.*, "Identification of Trace Volatile Compounds in Freshly Distilled Calvados and Cognac Using Preparative Separations Coupled with Gas Chromatography−Mass Spectrometry," *J. Agric. Food Chem.*, vol. 52, no. 16, pp. 5124–5134, Aug. 2004, doi: 10.1021/jf040052y.

[2] A. A. Apostolopoulou, A. I. Flouros, P. G. Demertzis, and K. Akrida-Demertzi, "Differences in concentration of principal volatile constituents in traditional Greek distillates," *Food Control*, vol. 16, no. 2, pp. 157–164, Feb. 2005, doi: 10.1016/j.foodcont.2004.01.005.

[3] O. Aguilar-Méndez *et al.*, "Volatile compound profile conferred to tequila beverage by maturation in recycled and regenerated white oak barrels from Quercus alba," *Eur. Food Res. Technol.*, vol. 243, no. 12, pp. 2073–2082, Dec. 2017, doi: 10.1007/s00217-017-2901-7.

[4] M. Caldeira, F. Rodrigues, R. Perestrelo, J. C. Marques, and J. S. Câmara, "Comparison of two extraction methods for evaluation of volatile constituents patterns in commercial whiskeysElucidation of the main odour-active compounds," *Talanta*, vol. 74, no. 1, pp. 78–90, Nov. 2007, doi: 10.1016/j.talanta.2007.05.029.

[5] A. Wanikawa, K. Hosoi, T. Kato, and K. Nakagawa, "Identification of green note compounds in malt whisky using multidimensional gas chromatography," *Flavour Fragr. J.*, vol. 17, no. 3, pp. 207–211, May 2002, doi: 10.1002/ffj.1073.

[6] P. Awad *et al.*, "Evolution of Volatile Compounds during the Distillation of Cognac Spirit," *J. Agric. Food Chem.*, vol. 65, no. 35, pp. 7736–7748, Sep. 2017, doi: 10.1021/acs.jafc.7b02406.

[7] J. A. Pino, S. Tolle, R. Gök, and P. Winterhalter, "Characterisation of odour-active compounds in aged rum," *Food Chem.*, vol. 132, no. 3, pp. 1436–1441, Jun. 2012, doi: 10.1016/j.foodchem.2011.11.133.

[8] J. Ledauphin, C. Le Milbeau, D. Barillier, and D. Hennequin, "Differences in the Volatile Compositions of French Labeled Brandies (Armagnac, Calvados, Cognac, and Mirabelle) Using GC-MS and PLS-DA," *J. Agric. Food Chem.*, vol. 58, no. 13, pp. 7782–7793, Jul. 2010, doi: 10.1021/jf9045667.

[9] L. Franitza, M. Granvogl, and P. Schieberle, "Characterization of the Key Aroma Compounds in Two Commercial Rums by Means of the Sensomics Approach," *J. Agric. Food Chem.*, vol. 64, no. 3, pp. 637–645, Jan. 2016, doi: 10.1021/acs.jafc.5b05426.

[10] M. D. C. A. de Souza, P. Vásquez, N. L. del Mastro, T. E. Acree, and E. H. Lavin, "Characterization of Cachaça and Rum Aroma," *J. Agric. Food Chem.*, vol. 54, no. 2, pp. 485–488, Jan. 2006, doi: 10.1021/jf0511190.

[11] I. Lukić, M. Banović, Đ. Peršurić, S. Radeka, and B. Sladonja, "Determination of volatile compounds in grape distillates by solid-phase extraction and gas chromatography," *J. Chromatogr. A*, vol. 1101, no. 1–2, pp. 238–244, Jan. 2006, doi: 10.1016/j.chroma.2005.09.057.

[12] S. C. Diéguez, Ma. L. G. de la Peña, and E. F. Gómez, "Approaches to Spirit Aroma: Contribution of Some Aromatic Compounds to the Primary Aroma in Samples of *Orujo* Spirits," *J. Agric. Food Chem.*, vol. 51, no. 25, pp. 7385–7390, Dec. 2003, doi: 10.1021/jf0302916.

[13] A. C. Garcia, F. A. T. Serafim, D. D. Keukeleire, and D. W. Franco, "Evaluation of Glycerol Profiles in Sugarcane Spirits (Cachaças)," *J. Braz. Chem. Soc.*, 2014, doi: 10.5935/0103-5053.20140213.

[14] L. M. Zacaroni *et al.*, "Determination of Phenolic Compounds and Coumarins in Sugar Cane Spirit Aged in Different Species of Wood," *Anal. Lett.*, vol. 44, no. 12, pp. 2061–2073, Aug. 2011, doi: 10.1080/00032719.2010.546017.

[15] E. Campo, J. Cacho, and V. Ferreira, "Solid phase extraction, multidimensional gas chromatography mass spectrometry determination of four novel aroma powerful ethyl esters," *J. Chromatogr. A*, vol. 1140, no. 1–2, pp. 180–188, Jan. 2007, doi: 10.1016/j.chroma.2006.11.036.

[16] E. Boothroyd, R. S. T. Linforth, F. Jack, and D. J. Cook, "Origins of the perceived nutty character of new-make malt whisky spirit: Nutty aroma of new make malt spirit," *J. Inst. Brew.*, vol. 120, no. 1, pp. 16–22, Jan. 2014, doi: 10.1002/jib.103.

[17] I. W. González-Robles and D. J. Cook, "The impact of maturation on concentrations of key odour active compounds which determine the aroma of tequila: Key odour active compounds which determine the aroma of tequila," *J. Inst. Brew.*, vol. 122, no. 3, pp. 369–380, Jul. 2016, doi: 10.1002/jib.333.
[18] P. Alberts, M. A. Stander, and A. De Villiers, "Development of a novel solid-phase extraction, LC-MS/MS method for the analysis of ethyl carbamate in alcoholic beverages: application to South African wine and spirits," *Food Addit. Contam. Part A*, vol. 28, no. 7, pp. 826–839, Jul. 2011, doi: 10.1080/19440049.2011.568010.
[19] J. A. Pino, "Characterization of rum using solid-phase microextraction with gas chromatography–mass spectrometry," *Food Chem.*, vol. 104, no. 1, pp. 421–428, Jan. 2007, doi: 10.1016/j.foodchem.2006.09.031.
[20] C. Da Porto, D. Decorti, and F. Tubaro, "Evaluation of volatile compounds and antioxidant capacity of some commercial rums from Dominican republic: Evaluation of volatile compounds and antioxidant capacity," *Int. J. Food Sci. Technol.*, vol. 46, no. 5, pp. 988–993, May 2011, doi: 10.1111/j.1365-2621.2011.02597.x.
[21] J. R. Belmonte-Sánchez *et al.*, "Rum classification using fingerprinting analysis of volatile fraction by headspace solid phase microextraction coupled to gas chromatography-mass spectrometry," *Talanta*, vol. 187, pp. 348–356, Sep. 2018, doi: 10.1016/j.talanta.2018.05.025.
[22] J. Demyttenaere, J. Sanchezmartinez, R. Verhe, P. Sandra, and N. Dekimpe, "Analysis of volatiles of malt whisky by solid-phase microextraction and stir bar sorptive extraction," *J. Chromatogr. A*, vol. 985, no. 1–2, pp. 221–232, Jan. 2003, doi: 10.1016/S0021-9673(02)01471-1.
[23] M. Caldeira, F. Rodrigues, R. Perestrelo, J. C. Marques, and J. S. Câmara, "Comparison of two extraction methods for evaluation of volatile constituents patterns in commercial whiskeysElucidation of the main odour-active compounds," *Talanta*, vol. 74, no. 1, pp. 78–90, Nov. 2007, doi: 10.1016/j.talanta.2007.05.029.
[24] J. S. Câmara *et al.*, "Comparative study of the whisky aroma profile based on headspace solid phase microextraction using different fibre coatings," *J. Chromatogr. A*, vol. 1150, no. 1–2, pp. 198–207, May 2007, doi: 10.1016/j.chroma.2006.09.014.
[25] S. Vichi, M. Riu-Aumatell, M. Mora-Pons, S. Buxaderas, and E. López-Tamames, "Characterization of Volatiles in Different Dry Gins," *J. Agric. Food Chem.*, vol. 53, no. 26, pp. 10154–10160, Dec. 2005, doi: 10.1021/jf058121b.
[26] P. Biernacka and W. Wardencki, "Volatile composition of raw spirits of different botanical origin: Volatile composition of raw spirits," *J. Inst. Brew.*, vol. 118, no. 4, pp. 393–400, Dec. 2012, doi: 10.1002/jib.55.
[27] M. Stupak, V. Kocourek, I. Kolouchova, and J. Hajslova, "Rapid approach for the determination of alcoholic strength and overall quality check of various spirit drinks and wines using GC–MS," *Food Control*, vol. 80, pp. 307–313, Oct. 2017, doi: 10.1016/j.foodcont.2017.05.008.
[28] M. Capobiango, R. B. Mastello, S.-T. Chin, E. de S. Oliveira, Z. de L. Cardeal, and P. J. Marriott, "Identification of aroma-active volatiles in banana Terra spirit using multidimensional gas chromatography with simultaneous mass spectrometry and olfactometry detection," *J. Chromatogr. A*, vol. 1388, pp. 227–235, Apr. 2015, doi: 10.1016/j.chroma.2015.02.029.
[29] Y. P. Zhao, X. P. Zheng, P. Song, Z. L. Sun, and T. T. Tian, "Characterization of Volatiles in the Six Most Well-Known Distilled Spirits," *J. Am. Soc. Brew. Chem.*, vol. 71, no. 3, pp. 161–169, Jun. 2013, doi: 10.1094/ASBCJ-2013-0625-01.

[30] J. V. Leland, P. Schieberle, A. Buettner, and T. E. Acree, Eds., *Gas Chromatography-Olfactometry: The State of the Art*, vol. 782. Washington, DC: American Chemical Society, 2001. doi: 10.1021/bk-2001-0782.

[31] Z. L. Cardeal and P. J. Marriott, “Comprehensive two-dimensional gas chromatography–mass spectrometry analysis and comparison of volatile organic compounds in Brazilian cachaça and selected spirits,” *Food Chem.*, vol. 112, no. 3, pp. 747–755, Feb. 2009, doi: 10.1016/j.foodchem.2008.06.057.

[32] A. De León-Rodríguez, L. González-Hernández, A. P. Barba de la Rosa, P. Escalante-Minakata, and M. G. López, “Characterization of Volatile Compounds of Mezcal, an Ethnic Alcoholic Beverage Obtained from *Agave salmiana*,” *J. Agric. Food Chem.*, vol. 54, no. 4, pp. 1337–1341, Feb. 2006, doi: 10.1021/jf052154+.

[33] J. Demyttenaere, J. Sanchezmartinez, R. Verhe, P. Sandra, and N. Dekimpe, “Analysis of volatiles of malt whisky by solid-phase microextraction and stir bar sorptive extraction,” *J. Chromatogr. A*, vol. 985, no. 1–2, pp. 221–232, Jan. 2003, doi: 10.1016/S0021-9673(02)01471-1.

[34] Y. Niu, D. Yu, Z. Xiao, J. Zhu, S. Song, and G. Zhu, “Use of Stir Bar Sorptive Extraction and Thermal Desorption for Gas Chromatography-Mass Spectrometry Characterization of Selected Volatile Compounds in Chinese Liquors,” *Food Anal. Methods*, vol. 8, no. 7, pp. 1771–1784, Aug. 2015, doi: 10.1007/s12161-014-0060-z.

[35] R. Delgado, E. Durán, R. Castro, R. Natera, and C. G. Barroso, “Development of a stir bar sorptive extraction method coupled to gas chromatography-mass spectrometry for the analysis of volatile compounds in Sherry brandy,” *Anal. Chim. Acta*, vol. 672, no. 1–2, pp. 130–136, Jul. 2010, doi: 10.1016/j.aca.2010.05.015.
